# Risk factors and treatment of pneumothorax secondary to granulomatosis with polyangiitis: a clinical analysis of 25 cases

**DOI:** 10.1186/s13019-018-0695-8

**Published:** 2018-01-15

**Authors:** Xuhua Shi, Yongfeng Zhang, Yuewu Lu

**Affiliations:** Department of Rheumatology and Immunology, Beijing Chao-Yang Hospital, Capital Medical University, 8 Gongren Tiyuchang Nanlu, Chaoyang District, Beijing, 100020 China

**Keywords:** Granulomatosis with Polyangiitis, Wegener’s granulomatosis, Pneumothorax

## Abstract

**Objectives:**

To investigate the risk factors and treatment strategies for pneumothorax secondary to granulomatosis with polyangiitis (GPA).

**Method:**

Retrospective analysis of cases with pneumothorax secondary to GPA from our own practice and published on literature.

**Results:**

A total of 25 patients, 18 males and 7 females, mean age 44 ± 15.7 years, were analyzed. Diagnosis included pneumothorax (11 cases), hydropneumothorax (*n* = 5), empyema (*n* = 8) and hemopneumothorax (*n* = 1). 88% (22/25) patients showed single/multiple pulmonary/ subpleural nodules with/without cavitation on chest imaging. Erythrocyte sedimentation rate and C-reactive protein were both elevated. Corticosteroids and immunosuppressive agents were used in 16 cases. Five cases received steroid pulse therapy, of which 4 patients survived. Pleural drainage was effective in some patients. Seven patients underwent surgical operations. In the 10 fatal cases, infection and respiratory failure were the most common cause. Lung biopsy/ autopsy showed lung/pleural necrotizing granulomatous vasculitis, breaking into the chest cavity, pleural fibrosis, bronchial pleural fistula, etc. The mean age in the death group was greater than the survival group (53 ± 12.9 years vs 40.1 ± 14.7 years, *p* = 0.05), the ineffective pleural drainage was also higher in the death group (5/5 vs 0/7, *p* = 0.01).

**Conclusions:**

Pneumothorax was seen in the active GPA, due to a variety of reasons, and gave rise to high fatality rate. Aggressive treatment of GPA can improve the prognosis. Older and lack of response for pleural drainage indicates poor prognosis.

## Background

**Fig. 1:**
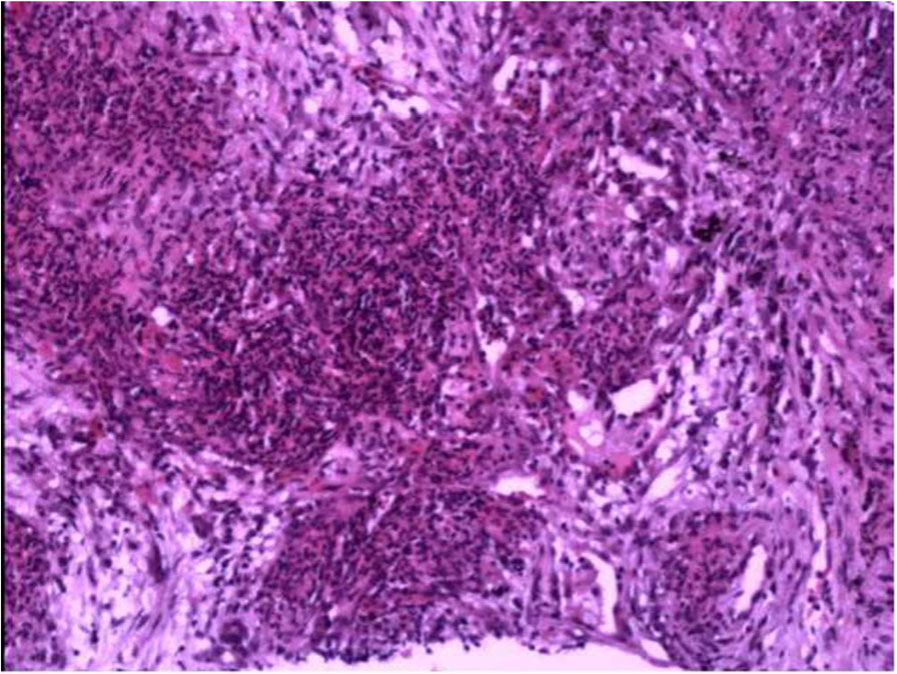
The microscopic features of pulmonary nodule obtained by percutaneous lung biopsy in a patient with granulomatous vasculitis (GPA). There are more infiltrating lymphocytes, neutrophils and eosinophils in fibrous tissues. Granulomas composed of tissue cells and inflammatory cells are also visible. There are prominent inflammatory cell infiltration and granuloma in the wall of small vessels. HE staining, 100×

Granulomatosis with polyangiitis (GPA), also known as Wegener’s granulomatosis, is a necrotizing granulomatous vasculitis, most commonly involving lung (Fig. 1), upper respiratory tract, and kidney [1]. When the respiratory system is involved, lung imaging study usually shows nodules, infiltrates, alveolar hemorrhage [2]. When pleural is involved, the patient can present with pleurisy, pleural effusion and thickening. Pneumothorax is a rare but serious complication, when occurred, can be categorized into pneumothorax, hydropneumothorax, empyema, hemopneumothorax, etc [3, 4]. So far there were only 20 cases of pneumothorax reported and the clinical presentation varied. Also, the cause and risk factors of GPA associated pneumothorax are unclear, and there is no consensus on important aspects of treatment strategy, such as, when the pneumothorax or infection is developed, how should treatment for the primary disease be adjusted? How to appropriately apply the pleural drainage and other surgical interventions? We encountered one case of GPA associated hydropneumothorax in our clinic who recovered after undergone active treatment of the primary disease and pleural drainage. The incidence of GPA associated pneumothorax is low. However, if not treated properly the fatality rate can be quite high. Currently there is still no cohort-based research. Therefore, we conducted a retrospective analysis of our case and all cases reported in the literature [4–24], aiming to identify the risk factors of GPA associated pneumothorax and explore the important principles of its clinical management once occurred, to guide the optimal treatment of the patients.

## Materials and methods

Because it is a retrospective research, the period of observation spans from 1978 (the first case) to 2015 (the last case). The patients came from two sources. The first was the case diagnosed and treated in our clinic. The second source included cases reported in the literature identified from the search of major databases, including: Chinese Biomedical Literature Database (CBM), Pubmed, EMBASE, and BIOSIS Preview. The searching key words were “pneumothorax”, “multi-vessel granulomatous inflammation” and “Wegener’s granulomatosis.” Full text articles were retrieved for review. All procedures performed in studies involving human participants were in accordance with the ethical standards of the institutional and/or national research committee and with the 1964 Helsinki declaration and its later amendments or comparable ethical standards.

Given the small number of reported cases and wide time span covered by the search, we went over the papers carefully, excluded the cases of pneumothorax unrelated to GPA (for example, pneumothorax caused by lung biopsy during the disease diagnosis). One lung imaging study included 31 cases of Wegener’s granulomatosis confirmed by clinical and pathology examination, but only had 1 case complicated by pneumothorax. We carefully reviewed and collected information on each patient’s clinical diagnoses, gender, age, time from disease onset to the development of pneumothorax, diagnosis of pneumothorax, the type of pneumothorax (pneumothorax, hydropneumothorax, empyema, hemopneumothorax), clinical presentations, laboratory test results (including anti-neutrophil cytoplasmic antibodies, anti-PR3 antibody, erythrocyte sedimentation rate, C-reactive protein), lung imaging findings (X ray / CT), corticosteroids and immunosuppressants treatment, pleural drainage, surgery, infection (infection site, pathogens, etc.), biopsy / autopsy pathology results. Four cases were reported from non-English literature, for which the information was gathered from the abstract written in English.

Case analysis included general patient characteristics (gender, age, duration of disease, type of pneumothorax, lung imaging findings, extrapulmonary manifestations, laboratory results, treatment and prognosis), infection (infection site, pathogens, etc.), biopsy / autopsy pathology results (lungs and other organs), as well as comparison between death and survived patients. This analysis included patients who met the 1990 American College of Rheumatology (ACR) classification criteria for Wegener’s granulomatosis [1, 25] defined as follows: 1) Nasal or oral inflammation: development of painful or painless oral ulcers or purulent or bloody nasal discharge; 2) Abnormal chest radiograph: chest radiograph showing the presence of nodules, fixed infiltrates, or cavities; 3) Urinary sediment: microhematuria (>5 red blood cells per high power field) or red cell casts in urine sediment; 4) Granulomatous inflammation on biopsy: histologic changes showing granulomatous inflammation within the wall of an artery or in the perivascular or extravascular area (artery or arteriole). Patients need to meet at least 2 criteria to be diagnosed as Wegener’s granulomatosis. The cases diagnosed before 1990 were confirmed with biopsy showing necrotizing granulomatous vasculitis.

### Statistical analysis

The general characteristics of the patients were summarized with descriptive statistics. Age, duration, anti-PR3, erythrocyte sedimentation rate (ESR) and C-reactive protein (CRP) were expressed as mean ± standard deviation, and were compared between death group and survival group using independent t-test. Categorical variables, including sex, types of pneumothorax, chest imaging (nodules, cavity, pulmonary hemorrhage), extrapulmonary manifestations (fever, skin lesions, nasal and sinus involvement, oral ulcers, renal involvement, nervous system involvement, arthralgia / arthritis, parotid swelling), positived ANCA, pleural drainage, surgery, spontaneous absorption, death, infection site, pathogens, biopsy site and pathology, therapy and interruption of treatment, risk factors for pneumothorax secondary to GPA, were summarized as percentage, and were analyzed between death group and survival group using chi-square test.

## Results

The first case in the series was from our clinic. The patient had sinusitis for 20 years, purulent nasal discharge, hemoptysis for 3 years, repeated skin ulcers for 2 years, difficulty in breathing for 2 months before being hospitalized. At admission, lung CT showed bilateral multiple nodules with/without cavity (biopsy indicated granulomatous inflammation). Right cavity ruptured and resulted in hydropneumothorax. C-ANCA was positive, the level of anti-PR3 was 150.5RU/ml. The condition improved after pleural drainage and treatment with corticosteroids and cyclophosphamide, cyclosporine A. The rest of the series included 1 case from the Chinese Biomedical Literature Database, and 23 cases from PubMed, EMBASE, BIOSIS Preview databases. The total was 25 cases.

The average age of the case series was 44 years, with a male to female ratio of 2.6: 1. The disease duration ranged from 1 month to 18 years. In 8 patients GPA was diagnosed after the occurrence of pneumothorax. The type of pneumothorax included pneumothorax, hydropneumothorax, empyema, hemopneumothorax. Respiratory symptoms presented were cough (5 cases), chest tightness and shortness of breath (9 cases), dyspnea (10 cases), and hemoptysis (2 cases). The majority of patients (88%) showed single or multiple nodules with/without cavitation on lung imaging. All patients had extrapulmonary manifestations, including fever, ear and nose involvement, neurologic symptoms, glomerulonephritis, and skin lesion. Most patients were treated with glucocorticoid (steroid pulse therapy for some patients) and immunosuppressive agents. In certain patients the medications were interrupted after the diagnosis of pneumothorax / infection and resulted in two deaths. None of the patients in the survivors group stopped the medication. Also, in the survivor group, about one-third patients received corticosteroid pulse therapy, while this was only 10% in the death group. Some patients did not undergo pleural drainage and successfully recovered from spontaneous absorption. The effects of pleural drainage were also mixed. Seven patients underwent surgeries, including pulmonary cavity resection, pulmonary lobectomy, and partial pleurectomy, etc. Four patients underwent surgery because the lung failed to re-expand or there were large persistent air volume leaks after pleural drainage and the details were not recorded properly. The fatality rate was high due to infection, respiratory failure, sepsis, respiratory arrest (Table 1). Infection involved the lungs, thoracic cavity, blood circulation, etc. Pseudomonas aeruginosa were frequently detected (Table 2). Biopsy/autopsy showed lung nodules, pleural necrotizing granulomatous vasculitis, breaking into the pleural cavity, pleural fibrosis, and bronchial pleural fistula (Table 3). The average age of death group was greater than the average age of survivor group (53 ± 12.9 years vs 40.1 ± 14.7 years, *p* = 0.05). In the death group, lack of response to pleural drainage which refers to the failure of the lung to re-expand or large persistent air volume leaks, was more common than that in the survivors (5/5 vs 0/7, *p* = 0.01) (Table 4).

**Table 1:** General characteristics of patients with pneumothorax secondary to GPA

Clinical features	Results
Male/Female (case)	18(72%)/7(28%)
Age(year)	44 ± 15.7(16~70)
Duration (weeks)	26 ± 51.0(0.83~216)
Pneumothorax type (case)	
Pneumothorax	11(44%)
hydropneumothorax	5 (20)
empyema^a^	8 (32%)
Hemopneumothorax	1 (4%)
Chest Imaging(n)	
Nodules (Multi/Single)	22(88%)
Cavity	21(84%)
Pulmonary hemorrhage	1 (4%)
Extrapulmonary manifestations (n)	
Fever	11(44%)
Skin lesions (purpura, gangrene, ulcers, etc.)	7 (28%)
Nasal and sinus involvement	15(60%)
Oral ulcers	5 (20%)
Glomerulonephritis and other	13(52%)
Nervous system (facial paralysis, mononeuropathy, etc.)	6 (24%)
Arthralgia/arthritis	11(44%)
Parotid swelling	2 (8%)
Laboratory tests	
ANCA-positive(n)	13/20(65%)
Anti-PR3(RU/ml)	176 ± 145.3(26~411)
ESR (mm/h)	92 ± 31.6(24~145)
CRP (mg/dl)	20 ± 27.4(2.35–90)
Pleural drainage(n)	16
Surgery(n)	7 (28%)
Spontaneous absorption(n)	4 (16%)
Death	10(40%)

Note: ANCA, anti-neutrophil cytoplasmic antibodies; anti-PR3, anti-proteinase 3 antibody; ESR, erythrocyte sedimentation rate; CRP, C-reactive protein

^a^These cases with empyema showed a clear pneumothorax at chest X-ray or CT scan

**Table 2:** Infection in patients with pneumothorax secondary to GPA

The site of infection and pathogens	*n* = 20
The site of infection	
Lung	4 (20%)
Pleural	9 (45%)
Blood	3 (15%)
Parotid	1 (5%)
Pathogens	
Pseudomonas aeruginosa	5 (25%)
Hemolytic streptococcus	2 (10%)
Proteus	1 (5%)
*Bacteroides fragilis*	1 (5%)
Streptococcus faecalis	1 (5%)
*Candida albicans*	1 (5%)
Aspergillus niger	1 (5%)

Note: A total of 20 cases

**Table 3:** Biopsy/autopsy results of patients with pneumothorax secondary to GPA

Biopsy site and pathology	Cases (n, %)
Lung (12 cases)	
Pulmonary nodules necrotizing granulomatous vasculitis	6/12(50.0%)
bronchial pleural fistula	2/12(16.7%)
Pleural necrotizing granulomatous vasculitis	1/12(8.3%)
Subpleural blister and fibrosis, pulmonary fibrosis and elastic tissue hyperplasia	1/12(8.3%)
Nose (necrotizing granulomatous vasculitis)	8
Skin (leukocytoclastic vasculitis)	2
Parotid (necrotizing granulomatous vasculitis)	1
Oral (necrotizing granulomatous vasculitis)	1

Note: lung biopsy/autopsy in a total of 12 cases

**Table 4:** Patients characteristics related to morality

Clinical features	Death group(*n* = 10)	Survival group	*P* value
		(*n* = 12)	
Male/Female(n)	7(70.0%)/3(30.0%)	8(80.0%)/4(40.0%)	1.000
Age (years)	53 ± 12.9(33~70)	40 ± 14.7(25~70)	0.050
Duration (weeks)	19 ± 24.8(10~48)	34 ± 69.2(0.83~216)	0.547
Extrapulmonary manifestations (n)			
Fever	3 (30.0%)	7 (58.3%)	0.231
Skin lesions (purpura, gangrene, ulceration)	3 (30.0%)	3 (25.0%)	1.000
Ear (hearing loss, otitis media)	2 (20.0%)	4 (33.3%)	0.646
Nasal and sinus involvement	5 (50.0%)	7 (58.3%)	1.000
Oral ulceration	4 (40.0%)	1 (8.3%)	0.135
glomerulonephritis	6 (60.0%)	6 (50.0%)	0.691
Nervous system (facial paralysis, multiple mononeuropathy, etc.)	3 (30.0%)	1 (8.3%)	0.293
Arthralgia/arthritis	4 (40.0%)	4 (33.3%)	1.000
Parotid swelling and pain	2 (20.0%)	0 (0.0%)	0.195
ESR(mm/h)	104 ± 26.7	82 ± 33.9	0.233
Infection(n)	6 (60.0%)	3 (25.0%)	0.192
Corticosteroids and Immunosuppressants (n)			
steroid pulse therapy	1 (10.0%)	4 (33.3%)	0.323
treatment interruption	2/4(50.0%)	0/8(0.0%)	0.091
Non-response to Pleural drainage (n)	5/5 (100%)	0/7 (0.0%)	0.010
Surgery(n)	2 (20.0%)	5 (41.7%)	0.381

Note: 3 cases without prognostic information, *n* = 22 cases; ESR, erythrocyte sedimentation rate

## Discussion

The incidence of pneumothorax in patients with GPA is low. So far, there were only individual case reports in the literature. Our analysis is the first systematic review of these cases. The results showed that pneumothorax secondary to GPA may be caused by many factors, is associated with high risk of fatality. Older age and non-response to pleural drainage indicate poor prognosis, and effective treatment of primary disease is very important to improve the prognosis.

**Fig. 2:**
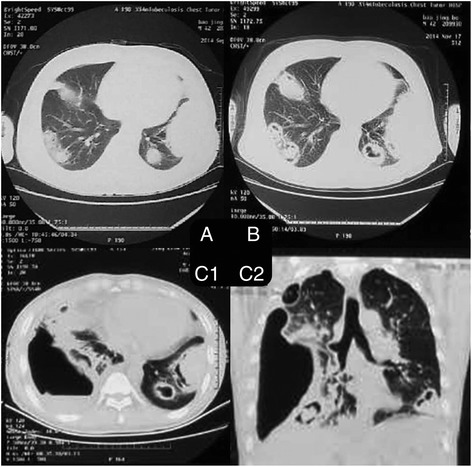
The bilateral fixed nodules, cavitation, right hydropneumothorax in a patient with granulomatous vasculitis (GPA)

There are different opinions for the cause of pneumothorax. Our analysis showed GPA associated pneumothorax is a collective effect of multiple factors (Table 5), rather than a single cause. First, 88% of patients in the analysis had pulmonary nodules, more frequent than the reported 40–70% in the general GPA patients [26]. Also, 21 out of 22 patients with pulmonary nodules had cavity, well above the 49% identified in general GPA patients population, [3] suggesting that breaking into the pleural cavity from cavitary nodule may be the main reason for the occurrence of pneumothorax (Fig. 2), especially cavitary nodules close to the pleura [9, 13, 17, 18]. Secondly, this analysis found that positive microbial culture of sputum and pleural drainage are common, and the same pathogen may be identified from both tests (in our patient, Aspergillus niger was detected from both sputum and pleural fluid drainage samples). This suggests that the secondary infection in the nodule cavity may contribute to the occurrence of pneumothorax, especially empyema in GPA patients [7]. The secondary infection is associated with cavity formation, and use of corticosteroids and immunosuppressants [8] not only increases the risk of infection, but also delays the wound healing after lung biopsy, thus causes pneumothorax [4]. Thirdly, the results of lung pathological examination in 2 patients showed bronchial pleural fistula is a pathological feature for pneumothorax [5, 8]. In addition, the disease progression itself involving the pleura may be a potential factor in the development of pneumothorax [3, 16]. In this analysis pleural biopsy revealed necrotizing granulomas and vasculitis. Epstein DM has reported a pneumothorax patient who did not have pulmonary manifestations at the diagnosis, but showed multiple pulmonary nodules one year later, suggesting pneumothorax may be caused by the primary disease itself that involves the pleura, and the emergence of pneumothorax indicates the likelihood of pulmonary lesion in the future [6]. Many patients in this analysis also had extrapulmonary manifestations. Inflammatory markers such as ESR, CRP were significantly increased, suggesting many of them were at the active stage of vasculitis when pneumothorax occurred. In only 1 patient who was confirmed GPA 18 years ago through nasal biopsy, wedge resection of lung and pleura partial resection were performed after the diagnosis of pneumothorax. Pathology exam showed subpleural blister with significant fibrosis, pulmonary fibroelastosis, no sign of vasculitis, and the pneumothorax may be caused by fibrous tissue traction [19]. Therefore, there are a few reasons and risk factors for pneumothorax in GPA patients. Breaking of nodule into the pleural cavity is the most common cause. Pneumothorax may occur at any time during the disease progression, while the majority of patients were in the active stage when it occurred.

**Table 5:** Risk factors for pneumothorax secondary to GPA

Risk factors	*N*
Active stage of Granulomatous vasculitis	19/20(95.0%)^a^
Pulmonary nodules broke into the chest cavity	15
Pleural necrotizing granulomatous vasculitis	1/12 (8.3%)^b^
Bronchial pleural fistula	2/12 (16.7%)^b^
Lesions fibrosis with pleural cohesion	1/12 (8.3%)^b^
Secondary infection in nodule cavity	3
Lesions fibrosis due to immunosuppressant	1
Delayed wound healing due to Glucocorticoid	2
Others	1

^a^Based on symptoms, laboratory tests, pathology

^b^Lung biopsy in 12 cases

This analysis also showed the prognosis of GPA patients complicated with pneumothorax is poor, and the fatality rate could be 40%, much higher than the general GPA patients [27]. The average age of patients who later died was higher than the survivors. Severe/ recurrent infections, respiratory failure were among the common causes of death. Lung infections, pleural cavity infections, blood infections are the most common forms of infection, and the most common pathogen is Pseudomonas aeruginosa. We also found, in certain patients the use of corticosteroids and immunosuppressive agents were interrupted or reduced after the development of pneumothorax/ infection with an intention to control the risk of infection [14]. However, this did not seem to improve the prognosis. We believe because most patients are in the active stage of vasculitis, the treatment for primary disease should be maintained and unnecessary interruption is not recommended [9]. Treatment of primary disease may increase the risk of infection. If infection is present, it is recommended that sensitive antibiotics be given besides the treatment of primary disease to improve the prognosis [17–19]. In addition, pleural drainage can remove pleural effusion and help lung recruitment, but our analysis showed that not all patients benefit from pleural drainage, especially in the death group where the procedure showed ineffective for all patients. Therefore, we speculate that lack of response to pleural drainage may be an indicator of poor prognosis. On the other hand, in some patients who did not undergo pleural drainage, pneumothorax was absorbed spontaneously after the treatment of primary disease. So, pleural drainage should be decided based on the situation of the patients. If the patient is in general good state and has no obvious respiratory symptoms, decreased oxygenation, pleural drainage may not be necessary [16]. If the patient’s condition is urgent, chest tube should be promptly placed. For patients not responding to pleural drainage, thoracotomy should be performed [21]. Also, for patients complicated with pleural infection, adequate drainage is essential [20]. Otherwise, extubation should be considered to reduce the risk of infection. Due to the small sample size, it is still unclear whether there are differences between different thoracic surgical methods. The common purpose of different surgical methods is to achieve lung expansion and to repair persistent air leaks. Therefore, video-assisted thoracic surgery (VATS) is recommended in order to reduce the damage to the patient. Not all nodules require operative resection except in those with cavity inside and adjacent to pleural. Similarly, pleurodesis is not always necessary and should be determined according to the patient’s actual conditions. According to my own understanding and experience, lung expansion and cessation of air leaks after surgical/immunosupressive therapy and the effective control of severe complications such as infection are most important factors for a better prognosis. Different surgical methods do not have significant impact on the prognosis.

In summary, pneumothorax secondary to GPA generally occurs during active phase of the disease, and is caused by a variety of reasons. Aggressive treatment of primary disease can improve the prognosis. Pleural drainage should be considered based on the condition of patients, and ineffective pleural drainage indicates poor prognosis. The incidence of pneumothorax secondary to GPA is low but the case fatality rate is high. Here we reported the first retrospective analysis of a large case series of GPA associated pneumothorax, provided a comprehensive review of the risk factors for pneumothorax, and analyzed the advantages and disadvantages of the main treatment approaches. We think the information can help clinicians identify high-risk patients for pneumothorax and make rational treatment decisions. Because this study was a retrospective analysis, limited to the number of cases reported in the literature, the results may not be generalizable to all GPA patients. Physicians should make decisions based on each patient’s condition.

## Conclusions

Pneumothorax was seen in the active GPA, due to a variety of reasons, and gave rise to high fatality rate. Aggressive treatment of GPA can improve the prognosis. Older and lack of response for pleural drainage indicates poor prognosis.

## Abbreviations

ACR: American College of Rheumatology
CBM: Chinese Biomedical Literature Database
CRP: C-reactive protein
ESR: Erythrocyte sedimentation rate
GPA: Granulomatosis with polyangiitis
